# Antibiotic course frequency and recovery strategies alter gut microbial composition and metabolism

**DOI:** 10.1093/ismeco/ycag145

**Published:** 2026-06-28

**Authors:** Kaoutar Abaakil, Zhigang Liu, Mu Wang, Elina Kuznecova, Ming S C Sung, Julian R Marchesi, Michaela A Mausz, Jia V Li

**Affiliations:** Division of Digestive Diseases, Department of Metabolism, Digestion and Reproduction, Faculty of Medicine, Imperial College London, London W12 0NN, United Kingdom; Division of Digestive Diseases, Department of Metabolism, Digestion and Reproduction, Faculty of Medicine, Imperial College London, London W12 0NN, United Kingdom; Luoyang Central Hospital Affiliated to Zhengzhou University, Luoyang, Henan 471000, China; Children' Hospital, Zhejiang University School of Medicine, National Clinical Research Center for Children and Adolescents' Health and Diseases, Hangzhou, Zhejiang 310052, China; Division of Digestive Diseases, Department of Metabolism, Digestion and Reproduction, Faculty of Medicine, Imperial College London, London W12 0NN, United Kingdom; Division of Digestive Diseases, Department of Metabolism, Digestion and Reproduction, Faculty of Medicine, Imperial College London, London W12 0NN, United Kingdom; Division of Digestive Diseases, Department of Metabolism, Digestion and Reproduction, Faculty of Medicine, Imperial College London, London W12 0NN, United Kingdom; Division of Digestive Diseases, Department of Metabolism, Digestion and Reproduction, Faculty of Medicine, Imperial College London, London W12 0NN, United Kingdom; School of Life Sciences, University of Warwick, Coventry CV4 7AL, United Kingdom; Division of Digestive Diseases, Department of Metabolism, Digestion and Reproduction, Faculty of Medicine, Imperial College London, London W12 0NN, United Kingdom

**Keywords:** metabolomics, ^1^H Nuclear Magnetic Resonance (NMR) spectroscopy, metabolic profiling, 16S rRNA gene sequencing, host-microbial interaction, antibiotics, small intestine

## Abstract

Antibiotics profoundly alter the gut microbiome, but how exposure frequencies shape microbial recovery remains unclear. The effectiveness of post-antibiotic interventions, e.g. probiotics or autologous fecal microbiota transplantation, (aFMT) requires further exploration. This study investigated how antibiotic course timing and recovery strategies influence gut microbiome and metabolism in male Wistar rats.

A single oral dose of vancomycin-ciprofloxacin (VC) caused rapid urinary and fecal metabolic shifts within 8–12 h and reduced bacterial α-diversity in cecal and colonic contents. When three VC courses were administered at regular (every 3 weeks; VCr) or irregular (1–3 weeks; VCi) intervals, VCr showed greater suppression of fecal α-diversity and stronger disruption of amino acid and host-microbial co-metabolism than VCi. Over the 3-week recovery period, VCr exhibited slower fecal α-diversity restoration; at week 3, β-diversity remained significantly different between groups, and cecal butyrate levels were persistently reduced in VCr. Both groups showed elevated levels of 5-aminovalerate in feces and colon compared with controls, whereas only VCi showed reductions in jejunal and ileal amino acids. Probiotics or aFMT had limited influence on small intestinal alterations, though aFMT accelerated fecal α-diversity recovery, and both interventions promoted partial normalization of fecal amino acids and 5-aminovalerate, without achieving complete restoration.

Overall, shorter antibiotic intervals exerted stronger effects on the small intestinal luminal chemical environment, whereas longer intervals led to greater suppression of colonic and fecal microbial metabolism. Probiotics and aFMT supported selective metabolic recovery without fully reversing antibiotics-induced disturbances, highlighting the need for more targeted restoration strategies across gastrointestinal regions.

## Introduction

Antibiotics remain crucial for treating life-threatening infections, yet their widespread use contributes to a growing global health crisis. Global antibiotic consumption increased by an estimated 46% between 2000 and 2018 [1], contributing to antimicrobial resistance, and elevating disease risks associated with the gut microbiome [2], which encompasses both microbial composition and metabolic function [3]. Even short-course antibiotic treatments can have lasting detrimental effects on microbial composition and function [4, 5]. The non-selective action of antibiotics depletes commensal gut bacteria, creating an environment susceptible to pathogen overgrowth and colonization [6]. Recovery from antibiotic-induced changes is often slow and incomplete [7, 8], resulting in long-term alterations to gut microbial communities and their associated metabolic activities. While the effects of antibiotic-induced dysbiosis have been extensively documented [7–9], the impact of varying antibiotic administration frequencies remains less understood. The frequency of antibiotic administration may critically shape the extent and dynamics of microbial and metabolic recovery. However, studies investigating this aspect are limited, leaving an important gap in our understanding of how different exposure regimens influence gut resilience.

Probiotics have been investigated for their potential to mitigate antibiotic-associated perturbations. While some studies report reductions in antibiotic-associated diarrhea [10], others show limited effects on restoring microbiome diversity [11]. Fecal microbiota transplantation (FMT), a method of transplanting the fecal microbiota from a healthy donor to a patient, has been shown as an effective treatment for patients with recurrent *Clostridioides difficile* infection [12]. FMT has also been explored as a method for restoring the gut microbiome in various diseases, such as obesity [13], diabetes [14], and antibiotics-associated diarrhea [15, 16]. While both probiotics and FMT have been explored as recovery strategies post-antibiotic treatment, their efficacy in restoring gut microbial diversity and composition is inconsistent [10, 11, 17]. These inconsistencies may be partially attributed to the reliance on fecal samples, which may not accurately reflect the diverse microbial environments throughout the gastrointestinal tract [18]. Furthermore, metabolic functions of the gut microbiota are key to maintaining a healthy gut environment. Antibiotics-induced changes extend beyond bacterial composition, influencing microbial metabolism and host-microbial co-metabolism, including reduced fecal short-chain fatty acids (SCFAs) and altered urinary metabolites, such as hippurate [19–21]. The observed shift from butyrate to glucose as the primary colonocyte energy source following antibiotic treatment highlights the impact of altered intestinal chemical environments on intestinal physiology [22].

In the current study, we investigated gut microbiome perturbations following three courses of vancomycin-ciprofloxacin (VC) administered at different frequencies. This combination, comprising vancomycin—a Gram-positive-targeting antibiotic [23] and ciprofloxacin—a broad-spectrum agent [24], provides profound suppression of bacterial abundance, diversity, and metabolic function. Previous research has demonstrated that 1-week course of vancomycin, ciprofloxacin, and metronidazole exerted profound and long-lasting effects on the fecal microbiome of healthy individuals [5]. Clinically, vancomycin and ciprofloxacin combination is an efficient first-line protocol antibiotic therapy for peritoneal dialysis-associated peritonitis [25]. In addition to characterizing microbiome disruptions, we explored the impact of recovery strategies, e.g. probiotics and FMT on restoring gut microbiome. To comprehensively assess the impact of antibiotic exposure frequency and recovery interventions, we employed both 16S rRNA gene sequencing and ^1^H nuclear magnetic resonance (NMR) spectroscopy-based metabolic phenotyping. This integrative approach enabled a systematic evaluation of changes in gut bacterial composition, intestinal luminal chemistry, and host–microbial metabolic interactions.

## Materials and methods

### Animal experiment

All animal work was performed under a Home Office UK issued license (P9718F9C8) adhering to animal welfare regulations. Fifty-eight outbred male Wistar rats, aged 6 weeks, were purchased from Charles River Laboratories and maintained under standard environmental conditions (22°C, 60%–70% humidity, 12 h/12 h day/night cycle). Single housing was implemented to minimize cross-contamination of microbiota between rats. A 2-week acclimatization period was applied before the start of the investigations. Standard rodent chow and water were provided *ad libitum* throughout the experiment. Body weight and food intake were recorded weekly and shown in Fig. S1. The three investigations were carried out as described below.

The single-dose experiment was conducted to evaluate dynamic changes induced by a single dose of VC blend. Five rats received a single oral dose of VC, while five control rats (CTL) received the drug vehicle (7% Tween 80 and 3% ethanol) at 8 a.m. *via* oral gavage. The VC blend consisted of vancomycin hydrochloride (50 mg/kg body weight) and ciprofloxacin hydrochloride (50 mg/kg body weight) dissolved in the drug vehicle as previously described [26]. Feces and urine were collected using metabolic cages at pre-treatment (0 h) and various post-treatment timepoints (0–4 h, 4–8 h, 8–12 h and 12–24 h). At 24 h, rats were euthanized using carbon dioxide inhalation and luminal content was collected from the duodenum, jejunum, ileum, cecum, and colon for bacterial profiling.

The long-term experiment was conducted to assess effects of VC administered at different frequencies. A total of 28 rats were randomly assigned to three groups: VCr (received VC at regular intervals of 3 weeks between two courses, *n* = 10), VCi (received VC at irregular intervals of 1 or 2 weeks between two courses, *n* = 10), and a drug vehicle-treated control group (7% Tween 80 and 3% ethanol, *n* = 8). Both VCr and VCi received three courses of VC (7 days/course), administered *via* oral gavage at 3 p.m. daily, using the doses described above. To match age and treatment frequencies, the control group was subdivided: four rats followed the regular schedule (CTLr) and four the irregular schedule (CTLi). Although each sub-control group consisted of only four rats, a statistical power of >80% was achievable using a two-sample *t-*test (two-tailed, α = 0.05), given the profound metabolic changes induced by oral antibiotics in the fecal and intestinal luminal environment. All animals underwent a 3-week recovery following the final VC course.

To evaluate recovery strategies following irregular VC administration, 20 rats underwent the same irregular VC treatment regimen as the VCi group. Two recovery interventions were implemented, including autologous fecal microbiota transplantation (aFMT, *n* = 10) and probiotic supplementation (*n* = 10). The aFMT group received a single dose of its own fecal microbiota via oral gavage 24 h after the end of each antibiotic course. The Probiotics group received daily oral gavage of probiotics throughout all recovery weeks. For FMT, two fecal pellets were dissolved in 1.2 ml of phosphate-buffered saline (PBS), vortexed for 3 min until thoroughly mixed. The solution was allowed to settle for 2 min, and 1 ml of the supernatant was administered to each rat by oral gavage. The “Supherb Bio-25” probiotic capsules, containing at least 25 billion active bacteria (*Bifidobacterium bifidum, Lacticaseibacillus rhamnosus, Lactococcus lactis, Lactobacillus casei subsp. casei, Bifidobacterium breve, Streptococcus thermophilus, Bifidobacterium longum subsp. longum, Lactobacillus casei subsp. paracasei, Lactiplantibacillus plantarum* and *Bifidobacterium longum subsp. infantis*), were used. Each capsule was dissolved in 20 ml PBS to achieve a final concentration of 1.25 billion bacteria/ml and a dosage of 4 × 10^9^ CFU/kg/day was administered.

Fecal and urinary samples were collected on the last day of each VC course (T1, T2, and T3) and at the end of each recovery week (R1, R2, and R3). The gut luminal contents were collected at the end of 3-week recovery. All samples were immediately placed on dry ice after collection and stored at −80°C.

### DNA extraction of feces and gut content, and Illumina sequencing

Bacterial DNA was extracted from 250 mg fecal pellets and gut luminal content using the QIAamp Power Fecal DNA kit (Qiagen, Crawley, UK) according to the manufacturer’s instructions. The extracted DNA was subsequently quantified using the Qubit 2.0 fluorometer (Thermo Fisher Scientific, USA) and diluted to 5 ng/ml. Sample libraries were prepared according to the Illumina 16S metagenomic sequencing library preparation protocol [27], including the steps of PCR amplification, library purification, indexing, library quantification, and normalization. The V1–V2 region of the 16S rRNA gene was amplified by PCR using primers 28F-YM, 28F-Borrellia, 28F-Chloroflex, 28F-Bifdo, and 388R. The V1–V2 region has been shown to have comparable performance with the V3–V5 region [28] and have a higher agreement with qPCR data for specific bacterial abundances compared to V3–V4 [29]. The Illumina MiSeq platform was employed to conduct sequencing in the Genomics Facility at the University of Warwick.

### Analysis of 16S rRNA gene-sequencing data

The sequences were pre-processed using the DADA2 package [30] within R (v.4.4.1). Amplicon sequence variants (ASVs) were assigned taxonomic labeling using a naïve Bayesian classifier and a SILVA v.128 training set. The data were analyzed using the phyloseq package in R (v.4.4.2). ASVs were filtered to remove low-abundance and low-prevalence features, excluding ASVs with a total count<4 across all samples and/or present in fewer than 5% of samples. Filtering was applied separately to the short-term and long-term datasets. The short-term dataset retained 772 ASVs (out of 4391) with a median sequencing depth of 32 278 reads per sample (IQR 26 430-37 631), while the long-term dataset retained 484 ASVs (out of 10 475) with a median sequencing depth of 23 676 reads per sample (IQR 19 032-28 929). α-diversity (Shannon and Simpson indices) was calculated at the ASV level using filtered, untransformed count data. β-diversity was assessed at the ASV level using weighted UniFrac distances calculated from total sum scaled relative abundance data and visualized using non-metric multidimensional scaling (NMDS). Differences in community composition were tested using permutational multivariate analysis of variance implemented via the adonis2 function from the vegan package.

For the single-dose experiment, differential abundance analysis was performed separately for each gut section using ANCOM-BC (ANCOMBC R package) at the family level on filtered raw count data. *P*-values were adjusted using the Benjamini–Hochberg (BH) procedure, and differentially abundant taxa were defined based on the ANCOM-BC decision criterion (diff_abn = TRUE) and an effect-size threshold of |natural log fold change| > 2. For the long-term antibiotic exposure experiment, longitudinal microbial dynamics were analyzed using SantaR (SantaR R package) on filtered raw count data from fecal samples, enabling comparison of treatment-associated temporal trajectories between experimental groups.

### ^1^H NMR spectroscopic analysis and spectral data analysis

Urine, feces, and gut content sample preparation and ^1^H NMR spectroscopic analyses are described in Supplementary Information. Mean-centered and unit variance-scaled spectral data were applied prior to multivariate statistical methods including unsupervised principal component analysis (PCA) and supervised orthogonal projection to latent structure discriminant analysis (OPLS-DA) with seven-fold cross-validation. OPLS-DA model validation was performed with a 1000-time permutation test.

### Integrals of ^1^H NMR spectral peaks and statistical analysis

Statistically significant peaks ensuing from OPLS-DA analysis were integrated using MATLAB (v. 2018b) to quantify relative concentrations of the identified fecal, urinary, and luminal metabolites. The chemical shifts for the metabolites included in this paper are outlined in Tables S1 and S2. The short asynchronous time-series analysis (SantaR) package was used in R (v.4.4.2) and RStudio (v. 2024.09.0 + 375) to compare time trajectories of fecal and urinary metabolites between different groups. *P*-values were calculated to determine if there were significant differences between group mean curves and corrected using the BH method (q values). Mixed-effects models were conducted in GraphPad Prism 9 software to compare gut luminal metabolite levels between groups and corrected using Tukey’s multiple comparisons tests. The “ComplexHeatmap” package was used to plot the log_10_-transformed ^1^H NMR spectral signal across different gut sections [31].

## Results

### Temporal changes in urinary and fecal metabolome within 24 h of vancomycin-ciprofloxacin treatment

**Figure 1:**
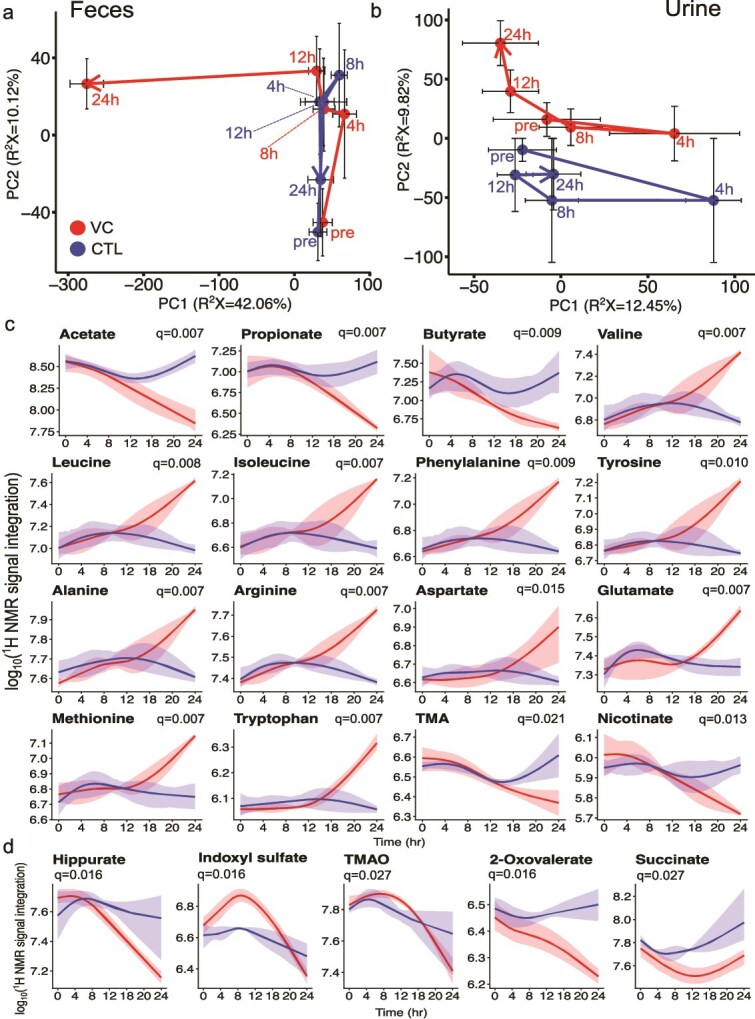
Temporal impact of VC on urinary and fecal metabolomes following a single oral dose. Trajectory PCA scores plots of ^1^H NMR spectral data from feces (a) and urine (b). Dots represent the mean of the scores of each treatment group at a time point, and error bars are standard error of the mean (SEM). Relative concentrations of fecal (c) and urinary (d) metabolites are plotted using log_10_ values of their representative ^1^H NMR peak integrals. Lines and shaded areas indicate mean curve and 95% confidence band of each group, respectively. BH-corrected *P*-values, q, indicate the statistical significance of the metabolic changes over time between VC and untreated control (CTL) groups. Key: PC, principal component; TMA, trimethylamine; TMAO, trimethylamine *N*-oxide.

We assessed fecal and urinary metabolic shifts within 24 h of VC treatment. Fecal profiles were stable for 12 h, but showed a marked shift along the first principal component (PC1) at 24 h (Fig. 1a). In contrast, urinary profiles diverged progressively from baseline and controls along PC2 (Fig. 1b). Relative concentrations of 16 fecal metabolites significantly altered post-VC. SCFAs (acetate, propionate, butyrate), trimethylamine (TMA), and nicotinate decreased, whereas amino acids (valine, leucine, isoleucine, phenylalanine, tyrosine, alanine, arginine, aspartate, glutamate, methionine, tryptophan) increased (Fig. 1c). Urinary hippurate, a host-microbial co-metabolite [32], declined from 12 h post-VC, indicating reduced microbial production of its precursor, benzoate. Indoxyl sulfate, a tryptophan-derived host-microbial co-metabolite [33], rose at 8–12 h, but dropped at 24 h (Fig. 1d). Relative concentrations of trimethylamine *N*-oxide (TMAO), produced from hepatic oxidation of TMA [33, 34], decreased at 24 h, consistent with reduced fecal levels of TMA. Succinate decreased from 8 h, and 2-oxovalerate from 12 h. Collectively, metabolic perturbations emerged in urine by 8–12 h and in feces by 12–24 h after a single VC dose.

### A single vancomycin-ciprofloxacin dose significantly disrupts bacterial composition in the large intestine within 24 h

**Figure 2:**
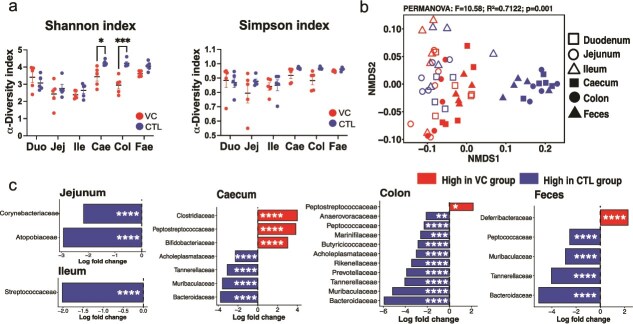
Impact of VC on the gut and fecal bacterial composition. α-diversity of bacterial communities in the duodenum (Duo), jejunum (Jej), ileum (Ile), cecum (Cae), colon (Col) and feces (Fae) (a). Data are expressed as mean ± SEM. *P*-values were calculated using two-way ANOVA between groups within each gut section and corrected using the BH procedure. β-diversity of the bacterial composition along the gastrointestinal tract is presented using NMDS scores plots (b). (c) Differentially abundant bacterial families identified using ANCOM-BC. Bars represent log fold change between VC and CTL groups across intestinal sections; only taxa with |log fold change| > 2 are shown. *P*-values were adjusted using the BH procedure. Red indicates enrichment in VC and blue indicated enrichment in CTL. ^*^P < .05, ^**^P < .01, ^***^P < .001, ^****^P < .0001.

VC significantly reduced the Shannon α-diversity of cecal and colonic bacterial communities within 24 h (Fig. 2a). β-diversity analysis showed distinct clustering of small and large intestine profiles from the control group, but this pattern was less pronounced in the VC group, indicating suppressive effects of VC on the bacterial abundances in the large intestine (Fig. 2b). VC significantly reduced jejunal *Corynebacteriaceae* and *Atopobiaceae* and ileal *Streptococcaceae*, together with *Bacteroidaceae, Muribaculaceae*, and *Tannerellaceae* abundances in the cecum, colon and feces (Fig. 2c). In contrast, *Bifidobacteriaceae, Peptostreptococcaceae, Clostridiaceae*, and *Deferribacteraceae* abundances were higher in the VC group in the cecum, colon, and feces.

### The frequency of antibiotic courses shapes fecal bacterial composition and its recovery trajectory

**Figure 3:**
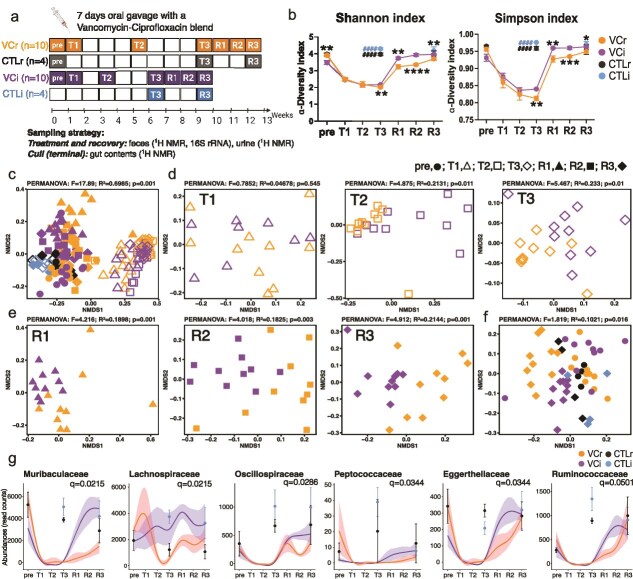
Impact of VC administration frequencies on the fecal bacterial composition. Experimental design and sample collection time points (a) created in BioRender. Abaakil, K. (2026) https://BioRender.com/muo23k7. α-diversity (b) are represented as mean ± SEM. Mixed-effects analysis was used with *post hoc* testing between groups for each time point, with *P*-values adjusted using the BH procedure (^*^P < .05, ^**^P < .01, ^***^P < .001, ^****^P < .0001). At T3 and R3, VC groups were compared to their corresponding control groups using two-way ANOVA with BH correction (^####^*P* < .0001). β-diversity is presented using NMDS scores plots with all samples (c) and at each time point (d–f). Lines and shaded areas indicate the mean trajectory of bacterial abundances and 95% confidence band of each group (g). The control groups are presented as mean ± SEM. BH-corrected *P*-values, q, indicate the statistical significance of differences in temporal trajectories between VCi and VCr groups.

To investigate whether the frequency of antibiotic administration influences the gut microbiome during the treatment and 3-week recovery, we compared rats receiving three courses of antibiotics at regular intervals (VCr) with those treated at irregular intervals (VCi) (Fig. 3a). As expected, antibiotic treatment significantly reduced Shannon and Simpson diversity indices, and at the end of the third course (T3), VCr showed significantly lower Shannon and Simpson diversity indices compared to VCi (Fig. 3b). During the recovery phase, VCr showed a slower recovery evidenced by lower α-diversity than VCi (Fig. 3b). Weighted UniFrac NMDS analysis showed that VC significantly disturbed the fecal bacterial composition (Fig. 3c) and VCr and VCi groups diverged at T2 and T3 (*P* = .01) (Fig. 3d). This difference between VCr and VCi remained across the recovery phase (Fig. 3e) with VCr group more distinct from the controls and pre-treatment profiles than VCi from its own controls (Fig. 3f). The recovery trajectories of *Muribaculaceae, Lachnospiraceae, Oscillospiraceae, Peptococcaceae*, and *Eggerthellaceae* were significantly different with a faster recovery in VCi (Fig. 3g). It is also worth noting that the abundance of the *Lachnospiraceae* family was not affected by VCi treatment.

### Antibiotic dosing frequency influences fecal and urinary metabolome and recovery trajectories

**Figure 4:**
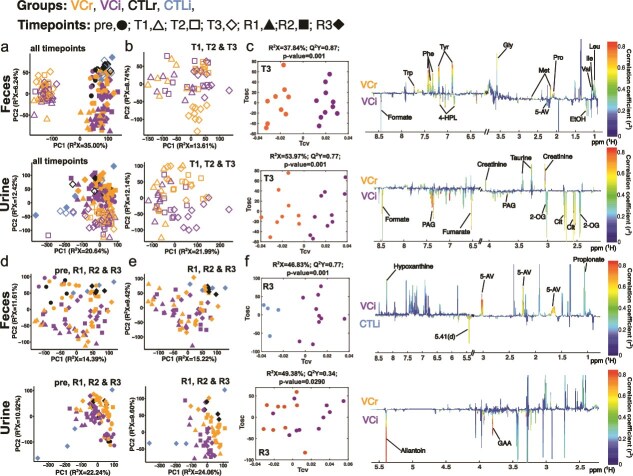
Impact of VC on the fecal and urinary metabolome. PCA scores plots of ^1^H NMR spectral data of feces and urine with all-time points (a), treatment time points only (b) and recovery (d&e). PC1 and PC2 are the first and second principal component, respectively, with the percentages indicating the variation explained by each component (R^2^X). OPLS-DA cross-validated scores and loadings plots of fecal and urinary ^1^H NMR spectral data at T3 (c) and R3 (f). Peaks pointing upwards represent higher relative concentrations of the metabolites in VCr group compared to VCi and vice versa, except for feces in (f), where peaks pointing upwards represent higher relative concentrations of the metabolites in VCi compared to CTLi. The colors of peaks indicate the correlation coefficient value square (r^2^). *P*-values of the model were derived from permutation tests with 1000 permutations. Key: 2-OG, 2-oxoglutarate; 4-HPL, 4-hydroxyphenyllactate; 5-AV, 5-aminovalerate; Cit, citrate; EtOH, ethanol; GAA, guanidoacetate; Gly, glycine; Ile, isoleucine; Leu, leucine; Met, methionine; PAG, phenylacetylglycine; Phe, phenylalanine; Pro, proline; Tau, taurine; Trp, tryptophan; Tyr, tyrosine; Val, valine.

We observed a clear separation between samples collected during antibiotic treatment (T1–T3) and those from the remaining time points, along PC1 in fecal profiles and PC2 in urinary profiles (Fig. 4a). Within the T1–T3 window, VCi and VCr groups were gradually separated in both fecal and urinary profiles (Fig. 4b) and statistically significant differences, assessed by OPLS-DA models, were observed in feces at T3 and urine from T2 (Table S3). Higher levels of fecal amino acids (glycine, methionine, tyrosine, phenylalanine, tryptophan, and branched-chain amino acids) were observed in VCr, whereas VCi showed elevated levels of formate, 5-aminovalerate (5-AV), 4-hydroxyphenyllactate and ethanol at T3 (Fig. 4c). These findings led us to conclude a stronger suppression of bacterial amino acid metabolism in VCr, supported by lower urinary levels of phenylacetylglycine (PAG), a phenylalanine-derived host-microbial co-metabolite, in the VCr group (Fig. 4c). Additionally, urinary levels of tricarboxylic acid (TCA) cycle intermediates (citrate, 2-oxoglutarate, and fumarate), and formate were higher in VCi than in VCr at T3 (Fig. 4c).

During the recovery phase, fecal profiles of VCr and VCi groups followed a similar trajectory (Fig. 4d and e). Although acetate levels were higher in the VCi group at R1 compared to VCr, no statistically significant differences were detected at R2 or R3 (Fig. S2A;  Table S3). In contrast to the control groups, both VCr and VCi led to persistently elevated fecal concentrations of 5-AV (Fig. 4f; Fig. S2b). In contrast, urinary profiles differed significantly between VCi and VCr across R1–R3, driven by higher concentrations of hippurate, allantoin, and guanidoacetate in VCi (Fig. S3a; Fig. 4f). It is worth noting that, in urine, no statistically significant difference was detected between VCr and its matched control group, CTLr, at R3, whereas differences remained between VCi and its matched controls, CTLi (Fig. S3B;  Table S3). The two control groups showed no metabolic differences in urine and feces (Table S3). The metabolite changes throughout the treatment and recovery phases are summarized in Fig. S4.

Together, these findings indicate that antibiotic dosing frequency (VCr vs. VCi) significantly shapes the fecal bacterial composition and metabolic profiles of urine and feces at the end of the third antibiotic course. However, this dosing frequency showed minimal impact on the trajectories of fecal metabolic profile recovery, but more frequent dosing (i.e. VCi) appears to prolong the recovery of urinary metabolic profiles.

### Metabolic landscape of the intestinal lumen following 3-week post-antibiotic recovery

**Figure 5:**
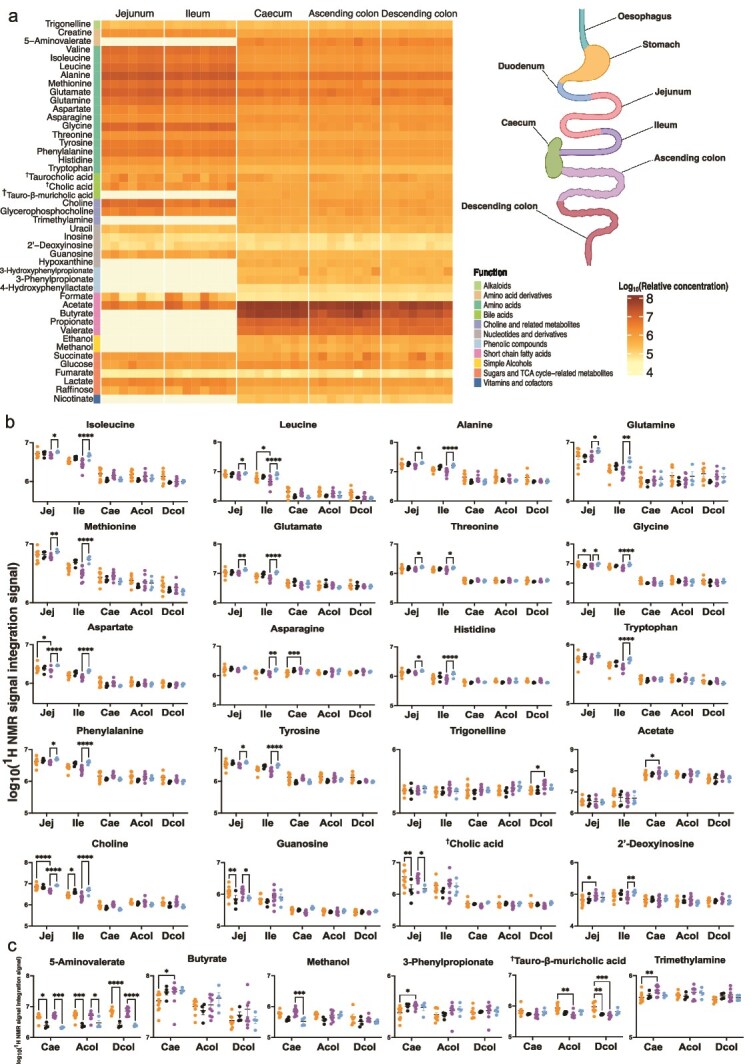
(a) Heatmap of relative concentrations of metabolites observed across the gastrointestinal tract of the non-antibiotic-treated control rats (CTLi, *n* = 4 and CTLr, *n* = 4). Logarithmic (log_10_) relative concentrations were calculated based on the area under the representative peaks of the metabolites in the ^1^H NMR spectra. Metabolites absent in upper gastrointestinal tract spectra were shaded with pale beige. (b–c) Log10-transformed ^1^H NMR spectral signal integrals, representing relative concentrations of metabolites in the gut content. Data are presented as mean ± SEM. *P*-values were calculated using two-way ANOVA *post hoc* testing between groups within each gut section and corrected using Tukey’s multiple comparisons test. ^*^P < .05, ^**^P < .01, ^***^P < .001, ^****^P < .0001. Key: Jej, jejunum; Ile, ileum; Cae, cecum; Acol, ascending colon; Dcol, descending colon. † Bile acids were putatively assigned based on a broad singlet in the methyl region (δ^1^H 0.60–0.72 ppm), corresponding to δ^1^H 0.72 (cholic acid), δ^1^H 0.67 (taurocholic acid) and δ^1^H 0.69 (tauro-β-muricholic acid).

In line with the spatial patterns observed for bacterial community composition across the GI tract (Fig. 2b), anatomical location was the predominant driver of variation in luminal metabolomes (Fig. S5a). Metabolic profiles from the small intestine (jejunum and ileum) were clearly distinct from those of the cecum and colon, largely due to higher amino acid concentrations in the small intestine (Fig. 5a). Duodenal data, analyzed separately owing to their low sample volume and different metabolite extraction dilutions, were unaffected by antibiotic treatment (Fig. S6).

Although luminal metabolic profiles were broadly similar between VCi and VCr, a greater number of metabolites differed significantly between VCi and its corresponding control (CTLi). In the small intestine, VCi group exhibited lower levels of amino acids (isoleucine, leucine, alanine, glutamine, glutamate, methionine, threonine, glycine, aspartate, asparagine, histidine, tryptophan, phenylalanine, tyrosine), choline, and 2′-deoxyinosine compared to CTLi, along with higher levels of bile acids (a singlet at 0.72 ppm putatively assigned as cholic acid) (Fig. 5b).

Several metabolites, including 5-AV, butyrate, methanol, 3-phenylpropionate, a singlet at 0.69 ppm (putatively assigned as tauro-β-murocholic acid) and TMA, were detected only in the cecal and colonic contents. Notably, cecal and colonic 5-AV levels were significantly elevated in both VCi and VCr compared to their controls (Fig. 5c), which are consistent with reported observations in feces (Fig. 4f). Cecal concentrations of butyrate, 3-phenylpropionate and TMA were significantly lower in VCr than in VCi, whereas putatively assigned tauro-β-murocholic acid in the colonic content was higher.

Taken together, these findings indicate that antibiotic dosing frequencies (VCi vs. VCr) influenced cecal and colonic metabolic environment even at the end of 3-week recovery. In contrast, VCi exerted stronger metabolic effects on the small intestinal environment compared to VCr.

### Autologous fecal microbiota transplantation and probiotics influence fecal bacterial compositional dynamics during antibiotic re-exposure and recovery

**Figure 6:**
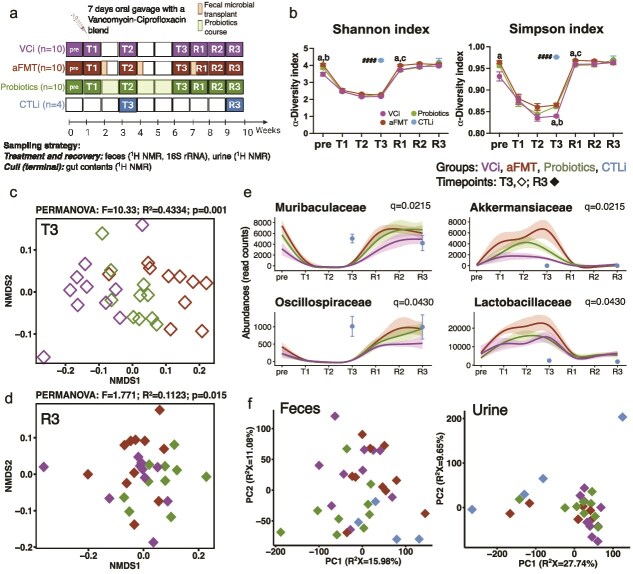
Impact of recovery strategies on the fecal bacterial composition and urinary and fecal metabolome. Experimental design and sample collection time points (a) created in BioRender. Abaakil, K. (2026) https://BioRender.com/az5ws3r. α-diversity of fecal bacterial profiles across antibiotic treatment and recovery are presented as mean ± SEM (b). Mixed-effects analysis was used for α-diversity with *post hoc* testing between groups for each time point, corrected using Tukey’s multiple comparisons test. *a* refers to significance (*P* < .05) between VCi vs aFMT, *b* between VCi vs Probiotics, *c* between aFMT and Probiotics. #### refers to significance (*P* < .0001) between each treatment group and control group (CTLi) determined by two-way ANOVA with BH correction. β-diversity of fecal bacterial profiles at T3 (c) and R3 (d) are presented using NMDS scores plots. Bacterial abundances are presented using mean trajectories (lines) and 95% confidence band of each group (shaded areas) (e). The control groups are presented as mean ± SEM. BH-corrected *P*-values, q, indicate the statistical significance in temporal trajectories between aFMT and VCi groups. No statistical significance was observed between Probiotics and VCi or aFMT. PCA scores plots of ^1^H NMR spectral data of feces and urine collected at R3 (f). PC1 and PC2 are the first and second principal component, respectively, with the percentages indicating the variation explained by each component (R^2^X).

We next investigated whether aFMT or probiotics influence the microbial and metabolic responses to subsequent antibiotic exposures and recovery (Fig. 6a). At T3, both aFMT and probiotic groups exhibited significantly higher Simpson diversity indices compared to the natural recovery group (VCi) (Fig. 6b) and clear grouping based on β-diversity (Fig. 6c), indicating that these interventions influenced fecal bacterial composition in response to subsequent antibiotic treatments. During the recovery, aFMT significantly enhanced α-diversity compared to VCi and Probiotics at R1, but no differences were observed at the later time-points (Fig. 6b). In contrast, β-diversity analysis revealed significant differences in fecal bacterial composition among VCi, aFMT and Probiotic groups (Fig. S7), and at R3 all three groups remained distinct from the control group (Fig. 6d). Fecal abundances of *Muribaculaceae* and *Oscillospiraceae* were markedly suppressed by antibiotics and showed faster recoveries to the control level in both aFMT and Probiotics groups (Fig. 6e). In contrast, *Akkermansiaceae* and *Lactobacillaceae* families increased, particularly in the aFMT group, in response to antibiotic treatment, and normalized during the recovery (Fig. 6e). These findings led us to conclude that aFMT exerted stronger effects than probiotics in shaping fecal bacterial composition during repeated antibiotic exposures and recovery.

### Autologous fecal microbiota transplantation and probiotics show selective impacts on the fecal, urinary, and gut luminal metabolome restoration

Despite these bacterial compositional shifts, we did not detect substantial metabolic impact on the global urinary or fecal profiles in response to Probiotics (Table S4; non-robust OPLS-DA models, permutation *P* > .05). Neither intervention fully restored the global fecal metabolomes to their pre-treatment state (Fig. S8a) or to the control group (Fig. 6f, Table S4). Both interventions, however, promoted a faster recovery of several fecal amino acid levels including tryptophan, phenylalanine, tyrosine, asparagine, aspartate, methionine, and leucine (Fig. S8b). Notably, aFMT, but not Probiotics, appeared to normalize the urinary metabolic profile towards the control group (Table S4, at R3, aFMT vs. CTLi *P* = .052; Probiotics vs. CTLi *P* = .011; VCi vs. CTLi *P* = .026).

**Figure 7:**
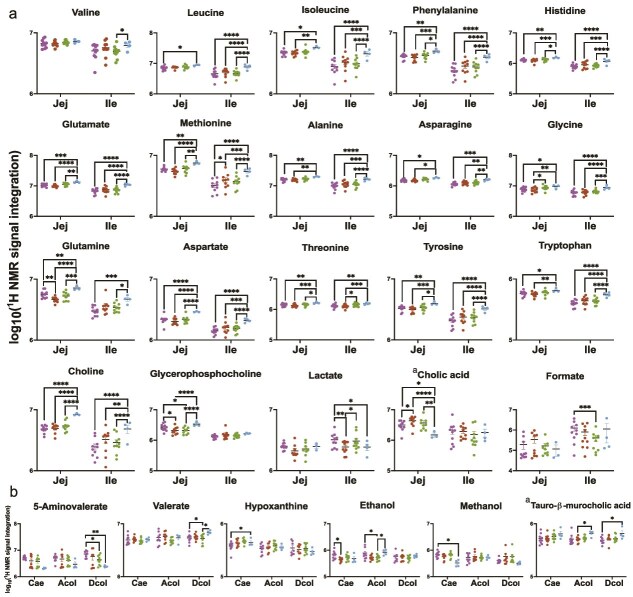
Impact of recovery strategies on gut content metabolic profiles. Relative concentrations of metabolites in jejunal (Jej) and ileal (Ile) contents (a), and in cecal (Cae), ascending colonic (Acol), and descending colonic (Dcol) contents (b) from the VCi (purple), aFMT (brown), Probiotics (green), and control (blue) groups. Data are represented as mean ± SEM. *P*-values were calculated using two-way ANOVA *post hoc* testing between groups within each gut section and corrected using Tukey’s multiple comparisons test. Significance stars were defined as: ^*^P < .05, ^**^P < .01, ^***^P < .001, ^****^P < .0001. Key: Jej, jejunum; Ile, ileum; Cae, cecum; Acol, ascending colon; Dcol, descending colon. ^a^Bile acids were putatively assigned based on a broad singlet in the methyl region (δ 0.60–0.72 ppm), corresponding to δ 0.72 (cholic acid) and δ 0.69 (tauro-β-muricholic acid).

We further explored the GI luminal metabolomes at R3, and no clear separation among aFMT, Probiotics, VCi and the control (CTLi) groups was noted in the PCA scores plots (Fig. S9). The univariate statistical analyses of relative concentrations of intestinal luminal metabolites showed that, compared to CTLi, levels of amino acids (e.g. leucine, isoleucine, phenylalanine, histidine, glutamate, methionine, alanine, aspartate, glycine, glutamine, threonine, tyrosine, tryptophan), along with choline and glycerophosphocholine, remained lower in the small intestine across all treatment groups (Fig. 7a). Lactate levels in the ileum normalized with aFMT, but remained elevated in natural recovery (Fig. 7a). In contrast, the putatively assigned cholic acid in the jejunum was not restored by either intervention (Fig. 7a). Probiotics specifically reduced ileal formate compared to VCi (Fig. 7a). In the colon, consistent with observations in the fecal metabolome (Fig. 4f), 5-AV remained elevated in the VCi group, whereas aFMT and Probiotics normalized its levels to near-control values (Fig. 7b). Levels of valerate, a fermentation product of amino acids [35], and ethanol in the colon appear to be suppressed by probiotic treatment, as evidenced by significantly lower levels compared with both the control and VCi groups (Fig. 7b). It is worth noting that although lower body weight was observed in the CTLi group (Fig. S1), it is unlikely to be a confounding factor, as CTLr and CTLi did not differ significantly in their urinary, fecal, and luminal metabolite profiles (Fig. 5b and c; Table S3).

## Discussion

### Temporal effects of a single vancomycin-ciprofloxacin dose

We reported that a single oral dose of VC significantly altered metabolic profiles of feces within 12–24 h and urine within 8–12 h. The observed metabolic changes are consistent with previous reports [21, 36]. VC reduced fecal SCFA levels, indicating suppressed anaerobic fermentation of complex carbohydrates [37]. In addition, VC increased fecal excretion of amino acids, which are typically released from protein hydrolysis by host endopeptidases and microbial proteases, and utilized by the gut microbiota to yield ammonia, phenols, indoles, amines, sulfides, and *N*-nitroso compounds [38]. Our observations suggested diminished microbial metabolism of amino acids, which were further supported by reduced levels of urinary host-microbial co-metabolites, such as hippurate and indoxyl sulfate, derived from bacterial metabolism of polyphenols and quinic acid [32], and tryptophan [33], respectively.

Interestingly, indoxyl sulfate levels initially rose at 8- and 12-h post-treatment before declining below control levels at 24 h. Its excretion is regulated in the kidney, and it is known to contribute to the progression of chronic kidney disease [39]. This excretion pattern could be associated with the temporal disturbances in the gut microbiota and/or renal responses to the VC treatment. Additionally, decreased fecal TMA and urinary TMAO levels point to disrupted microbial choline metabolism. Choline is an essential nutrient derived from phosphatidylcholine in animal sources and its microbial metabolism in the gut releases TMA and acetaldehyde [34]. TMA is absorbed through the hepatic portal system and subsequently oxidized to TMAO by flavin-containing monooxygenase-3 enzyme in the liver [33].

VC significantly perturbed bacterial composition in the cecum and colon, but not in the small intestine at 24 h post-treatment. This observation aligns with previous findings using a mixture of ampicillin, cefoperazone sodium salt, and clindamycin hydrochloride for 7 days [36]. Notably, bacterial shifts in feces did not fully mirror those in the intestinal lumen, suggesting luminal samples may better reflect antibiotic effects. The observations of reduced cecal and colonic abundances of *Tannerellaceae, Muribaculaceae*, and *Bacteroidaceae* and increased *Lactobacillaceae* post VC are consistent with published studies [36, 40–42]. Although vancomycin targets Gram-positive bacteria, *Lactobacillaceae* are often phenotypically resistant to its effect [43]. Furthermore, cecal *Clostridiaceae, Peptostreptococcaceae*, and *Bifidobacteriaceae* abundances increased following VC, supporting associations between increased abundances of *Clostridium* genus and short-term ciprofloxacin treatment [44].

### Antibiotic course frequency shapes gut microbial and metabolic recovery

We observed that longer recovery windows between antibiotic courses (VCr) resulted in a more pronounced suppression of colonic and fecal microbial metabolism, and lower fecal bacterial α-diversity compared with shorter intervals (VCi). This was reflected in higher relative concentrations of fecal amino acids and lower levels of urinary host-microbial co-metabolites, such as PAG. These findings align with previous reports that repeated antibiotic disturbances in humans can lead to persistent shifts in gut microbiota, even after initial recovery [8]. Such regime shifts, common in ecological systems, can result from repeated perturbations, preventing full restoration of community structure. The more substantial metabolic alterations observed in VCr support the presence of such functional regime shifts, highlighting the importance of antibiotic intake frequencies in preserving microbial and metabolic resilience. In addition, fecal abundances of *Muribaculaceae* were significantly lower in VCr, accompanied by lower cecal butyrate levels. Members of the *Muribaculaceae* (formerly S24-7) have been linked to butyrate production and high-fiber dietary intake [45–47], suggesting that VCr more strongly suppresses this potentially beneficial bacterial family. We also observed greater urinary loss of TCA cycle intermediates in the VCi group compared to VCr. Previous work has proposed that enhanced urinary excretion of TCA cycle intermediates may reduce substrate availability for lipogenesis [48], and we suggest that this finding is a potential systemic metabolic consequence of repeated antibiotic exposure. Further studies are needed to clarify the link between VCi and the reduced lipid accumulation relative to VCr.

Another profound observation was that shorter intervals between antibiotic courses exert stronger effects on the small intestinal luminal chemical environment, evidenced by significantly lower levels of amino acids in VCi compared to their corresponding untreated controls and this was not observed in comparison of VCr and their controls. These observations may imply increased absorption of amino acids in the small intestine following VCi, supported by previous evidence of upregulated protein expression levels of jejunal amino acid transporters (i.e. excitatory amino acid carrier 1 (EAAC1) and alanine, serine, cysteine transporter 2) and the peptide transporter (PepT1) and elevated circulating amino acid concentrations post-antibiotic treatment [49]. Furthermore, antibiotic treatment has been shown to reduce small intestinal and colonic motility [50], which may also contribute to increased absorption of amino acids. Alternatively, antibiotics may directly damage intestinal epithelial cells impairing metabolic processes as they have been shown to disrupt tight junction integrity which could facilitate passive loss of small molecules from the lumen into circulation [51, 52]. Although VCi was associated with faster recovery of fecal bacteria as observed in fecal microbiota analyses, the stronger metabolic effects observed in small intestine metabolome suggest that closely spaced antibiotic exposure may induce regional physiological disturbances not captured by fecal microbiota, potentially reflecting epithelial damage, motility changes or altered transporter activity that may not have sufficient time to recover between treatment courses. Notably, the reduced levels of amino acids in the small intestinal lumen were not restored using aFMT or probiotics post-antibiotic treatment; we suggest that these strategies have limited impact on the metabolome environment of the small intestine. These observations could be attributed to aFMT and probiotics containing bacteria that do not colonize in the small intestine [53] and/or their inability to reverse antibiotic-induced structural and functional epithelial changes.

In the cecum and colon, 5-AV concentrations in the luminal contents were significantly higher in VC-treated rats compared to untreated controls, regardless of the frequencies of antibiotic administration. 5-AV or 5-aminopentanoate can be produced from lysine via 5-aminopentanamide [54] and from D-proline by bacteria, including *C. difficile* [55]. With aFMT or probiotics, 5-AV levels were normalized but with a large intra-group variation compared to the control or VC-treated groups; we concluded that metabolic effects exerted by aFMT or probiotics in the large intestine varied drastically in individual animals and may be associated with colonization of the transplanted bacteria [56]. These colonized bacteria may promote the degradation of 5-AV to products such as valerate and ammonia [57]. Cecal luminal 5-AV in mice has been shown to positively correlate with colonocyte proliferation, which is a risk factor for colorectal cancer [58]. However, its biological function warrants further studies.

### Impact of autologous fecal microbiota transplantation and probiotics on the gut microbiome and metabolome recovery

aFMT promoted a faster recovery of fecal bacterial α-diversity compared to the probiotics-treated group. Our findings agree with previous studies, demonstrating a better recovery in bacterial composition with FMT compared to probiotics [17, 59]. Although aFMT and probiotics did not lead to a full recovery of the microbiome and metabolome, they promoted a quicker normalization of fecal amino acid levels. These observations align with the normalized concentrations of colonic luminal tyrosine and phenylalanine observed in patients with a *C. difficile* infection receiving FMT [60]. In addition, we observed that aFMT, but not probiotics, normalized the VCi-induced increase in ileal luminal lactate levels. Previous studies have shown that oral antibiotic treatment can shift host epithelial metabolism from oxidative metabolism to lactate fermentation, resulting in elevated luminal lactate [61]. Increased lactate availability may in turn enhance the risk of pathogen colonization, particularly when colonization resistance is weakened following antibiotic exposure [62]. aFMT appears to mitigate this risk by reducing lactate accumulation in the ileal lumen.

### Study limitations

While this study provides valuable insights, several limitations should be acknowledged. First, although the rat model offers a controlled system, it may not fully recapitulate the complexity and inter-individual variability of the human gut microbiota. Nonetheless, it remains informative, particularly for exploring core metabolic functions of the gut ecosystem. Second, the 3-week recovery period may not have been sufficient to capture full restoration of the microbiome and metabolome; extending the recovery duration in future studies would help determine whether these systems eventually return to a true baseline state. Additionally, the potential influence of the resistome, as well as dietary and age-related factors, which may shape microbiome recovery dynamics and metabolic outputs, was not assessed and warrants future investigation. Finally, further work examining additional recovery strategies, such as prebiotics or synbiotics, will be valuable for informing clinically relevant approaches.

## Conclusion

Antibiotic intake frequency had a profound impact on the gut microbiome and on metabolic activity across the gastrointestinal tract. Longer recovery intervals between courses led to more pronounced suppression of fecal bacterial diversity and microbial metabolism. Although both aFMT and probiotics facilitated partial metabolic normalization, neither approach fully restored microbial or metabolic function. These persistent disturbances may influence gut barrier integrity and homeostasis, potentially increasing susceptibility to gastrointestinal dysfunction. Furthermore, we reported significant luminal metabolomic disruptions associated with antibiotics in the small intestine, an often-overlooked region. Our findings highlight the importance of considering antibiotic dosing patterns and the recovery strategies employed between courses when evaluating post-antibiotic microbial and metabolic resilience. As aFMT and probiotics continue to be explored as therapeutic tools in microbiota-associated conditions, this work provides evidence to guide the development of more targeted and effective strategies for restoring gut microbial function after antibiotic exposure.

## Supplementary Material

ISME_Communications_Supplement-ZL_20260611

## Data Availability

^1^H NMR spectral data have been deposited to the MetaboLights data repository [63] under study number MTBLS12243. 16S rRNA gene-based sequencing data have been made available in the European Bioinformatics Institute (EBI) data repository under accession numbers PRJEB85923 (short-term study) and PRJEB85921 (long-term study).
