# Supplementary material for: Antibiotic course frequency and recovery strategies alter gut microbial composition and metabolism
Source: ISME Commun. 2026 Jun 28;6(1):ycag145. doi: 10.1093/ismeco/ycag145 (PMC13310174; doi:10.1093/ismeco/ycag145)
Supplement: ISME_Communications_Supplement-ZL_20260611 [file isme_communications_supplement-zl_20260611.docx]

**Supplementary Information**

**Antibiotic Course Frequency and Recovery Strategies Alter Gut Microbial Composition and Metabolism**

Kaoutar Abaakil^1,†^, Zhigang Liu^1,2,3,†^, Mu Wang^1^, Elina Kuznecova^1^, Ming S.C. Sung^1^, Julian R. Marchesi^1^, Michaela A. Mausz^4^, Jia V. Li^1,*^

^1^Division of Digestive Diseases, Department of Metabolism, Digestion and Reproduction, Faculty of Medicine, Imperial College London, London W12 0NN, United Kingdom

^2^Luoyang Central Hospital Affiliated to Zhengzhou University, Luoyang, Henan 471000, China

^3^Children’s Hospital, Zhejiang University School of Medicine, National Clinical Research Center for Children and Adolescents’ Health and Diseases, Hangzhou, Zhejiang 310052, China

^4^School of Life Sciences, University of Warwick, Coventry CV4 7AL, United Kingdom

^†^Kaoutar Abaakil and Zhigang Liu contributed equally to this work.

***Corresponding Author:** Jia V. Li, Section of Nutrition, Division of Digestive Diseases, Department of Metabolism, Digestion and Reproduction, Faculty of Medicine, Imperial College London, London W12 0NN, United Kingdom. Phone: +44 (0) 20 7594 3230; E-mail: jia.li@imperial.ac.uk.

**Materials and Methods**

**Preparation of urine, feces and gut content samples for ^1^H NMR spectroscopic analyses**

Fecal and gut luminal samples were weighed and extracted using high-performance liquid chromatography (HPLC)-grade water at a ratio of 1:3 (1 mg of sample to 3 μl of water) and 1:2 (1 mg of sample to 2 μl of water), respectively. The samples were vigorously vortexed, centrifuged at 20,000 *g* for 10 min and the supernatants were recovered. Urine samples were thawed at room temperature and mixed by vortexing for 5 s before centrifuging at 20,000 *g* for 10 min. A total of 540 μl of fecal and luminal aqueous extracts (excluding duodenal) or urine was mixed with 60 μl of 1.5 M potassium phosphate buffer made up of 100% D_2_O, pH 7.4, 1 mg/ml 3-(trimethylsilyl)-[2,2,3,3-^2^H_4_] propionic acid sodium salt (TSP) and 0.013 mg/ml sodium azide (NaN_3_, bacteriostatic reagent). A total of 580 μl of the mixtures were transferred to an NMR tube with an outer diameter of 5 mm. Due to the low sample volume, 180 μl of duodenal luminal extract was mixed with 20 μl of the potassium phosphate buffer with 180 μl of the total mixture transferred to an NMR tube with an outer diameter of 3 mm.

**^1^H NMR spectroscopic analysis**

^1^H NMR analysis was conducted with a Bruker DRX 600 MHz spectrometer (Bruker DRX, Rheinstetten, Germany) using a standard 1-dimensional pulse sequence [recycle delay (RD)-90°-t_1_-90°-t_m_-90°-free induction decay (FID) acquisition] at the temperature of 300 K. A total of 32 scans were collected into 64 K data points with a spectral width of 20 ppm. An RD irradiation of 4 s and a mixing time of 100 ms were used. A line broadening factor of 0.3 Hz was applied prior to Fourier transformation. Topspin (v 4.0) was used to automatically perform spectral phasing, baseline correction and calibration to the TSP peak (δ^1^H 0.0). Spectra within the δ1H 0-10 range were imported into MATLAB (version R2018b, The MathWorks, Inc., Natick, MA) with a resolution of 0.00035 ppm. Spectral regions covering TSP peak, water peak (δ^1^H 4.55-5.00) and urea peak (δ^1^H 5.535-6.075, for urinary spectra) were excluded. Spectral data were aligned to account for peak[1] shifts caused by variations in sample pH values and probabilistic quotient normalization[2] was performed. Statistical total correlation spectroscopy (STOCSY) [3] and Chenomx Software (Alberta, Canada) were used for metabolite identification.

**Table S1. Metabolites identified in feces and urine spectra from short-term single-dose experiment**

Key: d, doublet; dd, doublet of doublets; m, multiplet; q, quartet; s, singlet; t, triplet; bs, broad singlet

| **Metabolite** | **Biofluid** | **Chemical shifts (multiplicity)** | **Chemical shift range for peak integration (ppm)** |
| --- | --- | --- | --- |
| Acetate | Feces | 1.92(s) | 1.915-1.933 |
| Alanine | Feces | 1.48(d), 3.79(q) | 1.47-1.5 |
| Arginine | Feces | 1.66(m), 1.73(m), 1.92(m), 3.20(t), 3.77(t) | 1.712-1.753 |
| Aspartate | Feces | 2.69(dd), 2.80(dd), 3.89(dd) | 2.793-2.809 |
| Butyrate | Feces | 0.90(t), 1.56(m), 2.16(t) | 0.8831-0.8905 |
| Glutamate | Feces | 2.04(m), 2.12(m), 2.34(m), 3.75(m) | 2.337-2.376 |
| Isoleucine | Feces | 0.94(t), 1.01(d), 1.26(m), 1.48(m), 1.98(m), 3.68(d) | 1.015-1.023 |
| Leucine | Feces | 0.95(t), 1.71(m), 3.73(t) | 0.9602-0.9689 |
| Methionine | Feces | 2.14(s), 2.16(m), 2.65(t), 3.86(t) | 2.14-2.145 |
| Nicotinate | Feces | 7.53(dd), 8.26(dt), 8.61 (dd), 8.94(s) | 8.608-8.628 |
| Phenylalanine | Feces | 3.13(dd), 3.28(dd), 4.0(dd), 7.34(d), 7.39(m), 7.44(m) | 7.414-7.454 |
| Propionate | Feces | 1.06(t), 2.19(q) | 1.068-1.078 |
| Trimethylamine (TMA) | Feces | 2.88(s) | 2.874-2.886 |
| Tryptophan | Feces | 3.30(dd), 3.48(dd), 4.05(dd), 7.19(t), 7.27(t), 7.31(s), 7.53(d), 7.72(d) | 7.723-7.751 |
| Tyrosine | Feces | 3.06(dd), 3.20(dd), 3.94(dd), 6.89(d), 7.18(d) | 6.888-6.923 |
| Valine | Feces | 0.99(d), 1.04(d), 2.28(m), 3.62(d) | 0.9844-0.9921 |
| Hippurate | Urine | 3.97(d), 7.55(t), 7.64(t), 7.84(d) | 7.623-7.661 |
| Indoxyl sulfate | Urine | 7.20(t), 7.27(t), 7.36(s), 7.49(d), 7.72(d) | 7.687-7.712 |
| Trimethylamine *N*-Oxide (TMAO) | Urine | 3.27(s) | 3.276-3.28 |
| Succinate | Urine | 2.41(s) | 2.411-2.42 |
| 2-Oxovalerate | Urine | 2.70(t), 1.59(m), 0.91(t) | 0.9252-0.9289 |

**Table S2. Metabolites identified in different sample types from the long-term experiments**

Key: d, doublet; dd, doublet of doublets; m, multiplet; q, quartet; s, singlet; t, triplet; bs, broad singlet

**Table S3. Summary of parameters from OPLS-DA models of urinary and fecal ^1^H NMR profiles.** One OPLS component and one orthogonal component were fitted for each model and p values were derived from permutation analysis of 1000 permutations. F, feces; U, urine.

**Table S4. Summary of parameters from OPLS-DA models of urinary and fecal ^1^H NMR profiles from the recovery strategy experiment.** One OPLS component and one orthogonal component were fitted for each model and p values were derived from permutation analysis of 1000 permutations. F, feces; U, urine.

**Figure S1.** Average daily food intake per week (A) and body weight changes relative to the baseline (post acclimatization and prior to antibiotic treatment). T1-T3 and R1-R3 correspond to antibiotic-treatment courses and recovery periods, respectively (refer to the study design shown in Figures 3A and 6A). Food intake data (A) were analyzed using 2-way ANOVA (VCr *vs.* CTLr, non-significant; CTLi *vs.* the corresponding treatment groups, p<0.0001; aFMT *vs.* Probiotics/VCi, p=0.014). Body weight changes (B) were analyzed using simple linear regression and the slopes between groups were compared (CTLr *vs.* VCr, p=0.0001, CTLi *vs.* VCi/aFMT/Probiotics p<0.0001; VCi *vs.* aFMT *vs.* Probiotics, non-significant).

**Figure S2.** OPLS-DA cross-validated scores and loadings plots of fecal ^1^H NMR spectral data from VCr and VCi at R1 (A) and VCr and CTLr at R3 (B). Peaks pointing upwards represent higher relative concentrations of the metabolites in VCr group compared to VCi or CTLr and *vice versa*. The colors of the peaks indicate the squared correlation coefficient value (r^2^). P values of the model were derived from permutation tests with 1000 permutations. Key: 5-AV, 5-aminovalerate.

**Figure S3.** OPLS-DA cross-validated scores and loadings plots of urinary ^1^H NMR spectral data from VCr and VCi at R1-R3 (A) and from VCi and CTLi at R3 (B). Peaks pointing upwards represent higher relative concentrations of the metabolites in VCr group compared to VCi and *vice versa*. The colors of the peaks indicate the squared correlation coefficient value (r^2^). P values of the model were derived from permutation tests with 1000 permutations. ^†^Putatively assigned to dimethylglycine (DMG) based on a singlet at δ 2.93 ppm and STOCSY correlation with the singlet at δ 3.73 ppm.

**Figure S4.** Time trajectory plots of the logarithmic values of ^1^H NMR signal integrals of fecal (A) and urinary (B) metabolites. Lines and shaded areas indicate the mean time trajectory and confidence bands. q values are the Benjamini-Hochberg-corrected p values, indicating the statistical significance of the temporal trajectory over time between VCi (purple) and VCr (orange) groups. Key: 4-HPLA, 4-Hydroxyphenyllactate; PAG, Phenylacetylglycine. Mixed effects model with Benjamini-Hochberg correction was used to compare VCi and VCr at each time point. *p<0.05, **p<0.01, ***p<0.001; ****p<0.0001.

**Figure S5.** Principal component analysis (PCA) scores plots of ^1^H NMR spectra of the gut content collected from various sections of the gastrointestinal tract (A and B). PC1 and PC2 are the first and second principal components, respectively, with the percentages indicating the variation explained by each component (R^2^X).

**Figure S6.** Logarithmic values of ^1^H NMR spectral signal integrals, representing relative concentrations of metabolites in the duodenal content collected from regular and irregular VC-treated rats (VCr, VCi) and their corresponding untreated controls (CTLr and CTLi). Data are presented as mean ± SEM. No statistically significant differences were observed using one-way ANOVA with post hoc testing between groups.

**Figure S7.** β-diversity presented using non-metric multidimensional scaling (NMDS) scores plots of fecal bacterial profiles of VCi, aFMT, and probiotics groups during recovery weeks. PERMANOVA analysis was used with: R1: F-value: 2.2649; R-squared: 0.14837; p-value: 0.008, R2: F-value: 1.9142; R-squared: 0.12418; p-value: 0.013, R3: F-value: 2.0432; R-squared: 0.13145; p-value: 0.003.

**Figure S8. (A)** Principal component analysis (PCA) scores plots of ^1^H NMR spectra of feces and urine collected from VCi, aFMT and Probiotics groups before antibiotic treatment (pre) and across the recovery (R1-R3), along with the corresponding control group (CTLi) at R3. PC1 and PC2 are the first and second principal components, respectively, with the percentages indicating the variation explained by each component (R^2^X). Time trajectory plots of the logarithm values of ^1^H NMR signal integrals of fecal (B) and urinary (C) metabolites. Lines and shaded area indicate the mean time trajectory and 95% confidence bands. No metabolite trends across time points between groups are statistically significant based on Benjamini-Hochberg-corrected p values. Mixed effects model with Benjamini-Hochberg correction was used to compare metabolite levels across three groups at each time point. a refers to significant differences between VCi and aFMT, b between VCi and probiotics, and c between aFMT and probiotics. Key: 4-HPLA, 4-Hydroxyphenyllactate; 5-AV, 5-aminovalerate; PAG, Phenylacetylglycine. ^†^Putatively assigned to dimethylglycine based on a singlet at δ 2.93 ppm and STOCSY correlation with the singlet at δ 3.73 ppm.

**Figure S9.** Principal component analysis (PCA) scores plots of ^1^H NMR spectra of the gut content collected from the duodenum, jejunum, ileum, caecum, ascending colon and descending colon (A and B). PC1 and PC2 are the first and second principal components, respectively, with the percentages indicating the variation explained by each component (R^2^X).

**References**

1. Veselkov KA, Lindon JC, Ebbels TM *et al.* Recursive segment-wise peak alignment of biological (1) H NMR spectra for improved metabolic biomarker recovery. *Anal Chem*. 2009;**81**:56-66 <https://doi.org/10.1021/ac8011544>

2. Dieterle F, Ross A, Schlotterbeck G *et al.* Probabilistic quotient normalization as robust method to account for dilution of complex biological mixtures. Application in 1H NMR metabonomics. *Anal Chem*. 2006;**78**:4281-90 <https://doi.org/10.1021/ac051632c>

3. Cloarec O, Dumas ME, Craig A *et al.* Statistical total correlation spectroscopy: An exploratory approach for latent biomarker identification from metabolic 1H NMR data sets. *Anal Chem*. 2005;**77**:1282-9 <https://doi.org/10.1021/ac048630x>
